# Elevated expression of CK19, Ki67, and β-Catenin as prognostic biomarkers in hepatocellular carcinoma

**DOI:** 10.1186/s12885-025-15429-6

**Published:** 2025-12-12

**Authors:** Hongjiu Yu, Jiaying Wu, Lianghui Gao

**Affiliations:** Key Laboratory of Emergency and Trauma of Ministry of Education, Departmentof Hepatopancreatobiliary surgery ,The First Affiliated Hospital, Hainan Medical University, Haikou, China

**Keywords:** CK19, Ki67, Β-catenin, Hepatocellular carcinoma, Survival analysis

## Abstract

**Background:**

Cytokeratin 19 (CK19), Ki67 antigen (Ki67), and β-catenin are abnormally overexpressed in hepatocellular carcinoma (HCC), but their diagnostic and prognostic value remains unclear. This study aims to investigate the predictive role of these three markers in post-operative survival of HCC patients.

**Methods:**

The expression levels of CK19, Ki67, and β-catenin in HCC tumor tissues were determined through public datasets. Kaplan-Meier survival analysis and multivariate Cox regression were performed to evaluate their prognostic value. Immunohistochemistry and Western blotting were used to detect the expression levels of CK19, Ki67, and β-catenin in hepatocellular carcinoma tissues and adjacent non-cancerous tissues. Transcriptome sequencing was performed to analyze the differential transcriptional changes between HCC and adjacent non-cancerous tissues. A cohort of HCC post-operative patients was included to analyze the correlation between the three markers and clinical pathological features.

**Results:**

CK19, Ki67, and β-catenin were highly expressed in HCC tissues and lowly expressed in adjacent non-cancerous tissues. High expression of CK19, Ki67, and β-catenin was closely associated with poor disease-free survival (DFS) and overall survival (OS) in HCC patients’ post-surgery. These three markers serve as independent prognostic factors for DFS and OS. Immunohistochemistry and Western blotting revealed upregulation of CK19, Ki67, and β-catenin in HCC tissues, while transcriptome sequencing indicated alterations related to metabolic reprogramming, immune evasion, and invasion/metastasis in HCC. Clinical data from HCC patients showed that CK19 expression correlated with tumor number and differentiation grade, Ki67 expression correlated with patient age, tumor size, tumor number, lymphatic metastasis, and tumor differentiation, while β-catenin expression was closely related to tumor diameter, number, and capsule status.

**Conclusion:**

CK19, Ki67, and β-catenin are highly expressed in HCC and can serve as molecular markers for post-operative recurrence and poor survival in HCC patients, providing a basis for precise prognostic evaluation in HCC.

**Supplementary Information:**

The online version contains supplementary material available at 10.1186/s12885-025-15429-6.

## Introduction

Hepatocellular carcinoma (HCC) is one of the most common malignancies globally, particularly in Asia and Africa, with its high incidence closely associated with factors such as liver cirrhosis, chronic hepatitis B and C virus infections, and alcoholic liver disease [1]. Additionally, the occurrence of HCC is also closely related to genetic, environmental, lifestyle factors, and metabolic diseases [2]. The clinical presentation of HCC is diverse, and early-stage HCC often lacks obvious symptoms, which leads many patients to be diagnosed at an advanced stage. The main treatment options for HCC include surgical resection, liver transplantation, local ablation, chemotherapy, radiotherapy, and targeted therapy [3]. Among these, HCC radical surgery is one of the most effective treatment methods for early-stage HCC, significantly improving the five-year survival rate of patients [4, 5]. However, due to the high risk of early (within two years after surgical resection) HCC recurrence, the survival rate of HCC patients remains low. Notably, commonly used tumor markers such as serum alpha-fetoprotein (AFP) are ineffective in predicting early HCC recurrence [6]. The sensitivity of traditional AFP markers for early recurrence prediction is only 40%–60%, and its ability to assess recurrence risk in patients with normal AFP levels is limited [7]. Therefore, the discovery of more robust and effective early HCC recurrence biomarkers is crucial for improving the treatment outcomes of HCC patients. In recent years, with the advancement of molecular pathology and tumor microenvironment research, biomarkers such as cytokeratin 19 (CK19), Ki67 antigen (Ki67), and β-catenin have gradually become key targets for analyzing HCC heterogeneity, invasiveness, and recurrence risk. Their critical roles in tumor stem cell characteristics, proliferation dynamics, and signaling pathway regulation have garnered significant attention [8–10].

In HCC, CK19, as a key marker for cancer stem cells, is closely related to the tumor’s heterogeneity and invasiveness [11, 12]. The formation of the CK19-positive phenotype in HCC may be driven by tumor microenvironment-mediated transformation of CK19-negative cell phenotypes. These transformed cells, with enhanced self-renewal ability and abnormal differentiation potential, further promote tumor malignant progression [12]. Clinical data show that 4–28% of HCC patients exhibit CK19-positive expression, and these cases are often accompanied by high AFP expression, hepatitis B virus positivity, and more aggressive biological behavior, leading to significantly reduced overall survival and recurrence-free survival (RFS) rates [13, 14]. Although the World Health Organization has listed CK19 as a prognostic marker for HCC, its clinical definition standard is not yet unified. Investigating the relationship between CK19 expression characteristics and clinical features is of great clinical value for achieving precise stratified management of patients.

In the molecular pathological evaluation of HCC, Ki67 antigen, as a core marker of cellular proliferation activity, reflects tumor malignancy through its sustained expression in the cell cycle [15]. Research has shown that high expression of Ki67 is closely related to HCC cell cycle dysregulation and genomic instability [16]. Clinical observations indicate that high Ki67 expression is associated with significantly shortened post-operative recurrence-free survival, as well as reduced response to targeted therapy, suggesting its prognostic predictive value. Notably, the spatial heterogeneity of Ki67 expression at the tumor margin is correlated with microvascular invasion risk, providing a potential molecular basis for assessing surgical margins [17]. Additionally, dynamic expression characteristics of Ki67 are being integrated into HCC molecular subtyping systems, offering new research directions for optimizing individualized recurrence monitoring and precise treatment strategies.

As a pivotal molecule in the Wnt/β-catenin signaling pathway, β-catenin gene mutations or abnormal activation are present in 30%−40% of HCC cases, and its dysfunction has become a key molecular event in HCC development [18]. Studies have shown that β-catenin activates the transcriptional programs of oncogenes such as c-Myc and Cyclin D1 through nuclear translocation, while upregulating the expression of epithelial-mesenchymal transition markers such as vimentin and Snail, thereby endowing tumor cells with invasive phenotypes [19, 20]. In vivo and in vitro experiments have confirmed that β-catenin-activated HCC models exhibit a significant tendency for vascular invasion, higher satellite nodule formation rates compared to wild-type models, and an increased risk of recurrence within 12 months post-surgery [21].

In summary, CK19, Ki67, and β-catenin reveal the molecular basis for HCC recurrence from the perspectives of stem cell characteristics, proliferation dynamics, and signal transduction. However, the clinicopathological relevance of CK19, Ki67, and β-catenin in HCC remain unclear. Therefore, this study aimed to systematically characterize the expression profiles and prognostic implications of these markers by integrating bioinformatics analysis, immunohistochemistry, transcriptomic sequencing, and clinicopathological correlation studies. Analyzing the expression patterns, interaction networks, and dynamic associations with clinical features of these markers will provide theoretical support for the establishment of multi-dimensional prognostic models and the development of targeted recurrence monitoring strategies. This study aims to retrospectively analyze the clinical and pathological data of 110 HCC patients, investigate the relationship between CK19, Ki67, and β-catenin expression and clinical pathological features, and preliminarily analyze their association with HCC post-operative recurrence to explore the clinical significance of these three markers in HCC.

## Materials and methods

### Database analysis

The TIMER 2.0 database (https://cistrome.shinyapps.io/timer/) and GEPIA database (http://gepia.cancer-pku.cn/) were used to evaluate the expression levels of CK19, Ki67, and β-catenin in HCC. Additionally, the GEPIA database and Kaplan-Meier Plotter (http://kmplot.com/analysis) were used to analyze the associations between the expression of CK19, Ki67, and β-catenin and patient survival outcomes, including overall survival (OS), recurrence-free survival (RFS), progression-free survival (PFS), and disease-specific survival (DSS).

### Clinical data of HCC patients

This study enrolled 110 patients who underwent surgical treatment for HCC at the First Affiliated Hospital of Hainan Medical University. Clinical data and a portion of surgical tissue samples were collected. Clinical information included gender, age, tumor size, tumor number, TNM stage, presence of cirrhosis, vascular invasion, serum AFP levels, lymph node metastasis, and tumor differentiation. Tissue samples included both tumor and adjacent non-tumorous liver tissues. Patients were followed up monthly during the first six months after surgery, and every three months thereafter, with recurrence monitored by imaging studies (CT and MRI) and serum AFP measurements. Informed consent was obtained from all patients, and the study was approved by the Ethics Committee of the First Affiliated Hospital of Hainan Medical University.

### Immunohistochemistry (IHC)

Formalin-fixed, paraffin-embedded tissue sections were prepared and baked at 65 °C for 2 h. Sections were deparaffinized in xylene and rehydrated through a graded series of ethanol. Antigen retrieval was performed using 0.01 M citrate buffer (pH 6.0), followed by overnight incubation at 4 °C with primary antibodies against CK19 (1:100), Ki67 (1:100), and β-catenin (1:100). After washing, the sections were incubated with horseradish peroxidase (HRP)-conjugated secondary antibodies at room temperature for 30 min. Visualization was achieved with diaminobenzidine (DAB) solution and counterstaining with hematoxylin. Staining results were evaluated based on staining intensity and the percentage of positive cells. Staining intensity was scored as: 0 (negative), 1 (weak), 2 (moderate), and 3 (strong). The percentage of positive cells was categorized into five grades: Grade 0 (0%), Grade 1 (1–25%), Grade 2 (26–50%), Grade 3 (51–75%), and Grade 4 (76–100%). The final immunohistochemical score was obtained by multiplying the intensity score by the percentage score, resulting in four expression levels: 0 (negative), 1–4 (weak), 6–8 (moderate), and 9–12 (strong). All samples were independently evaluated by two blinded pathologists using the scoring system described above.

### Western blotting (WB)

Tumor and adjacent non-tumorous tissues were lysed on ice for 30 min using RIPA buffer supplemented with protease inhibitors. Total protein was extracted by centrifugation at 12,000 × g for 15 min. Protein concentration was determined using the BCA assay. Equal amounts of protein (30 µg) were mixed with SDS-PAGE loading buffer, denatured at 95 °C for 5 min, and separated by SDS-PAGE (5% stacking gel, 10% separating gel, 80 V until entering the separating gel, then 120 V for separation). Proteins were transferred onto activated PVDF membranes at 300 mA constant current for 90 min. Transfer efficiency was confirmed using a pre-stained protein marker. Membranes were blocked with 5% non-fat milk at room temperature for 1 h, then incubated overnight at 4 °C with primary antibodies (anti-CK19, anti-Ki67, and anti-β-catenin, all at 1:1000 dilution). After washing with TBST, membranes were incubated with HRP-conjugated secondary antibodies (1:5000 dilution) at room temperature for 1 h. Protein bands were visualized using an ECL chemiluminescence kit and imaged using a chemiluminescence detection system. Band intensities were quantified using ImageJ software.

### RNA sequencing (RNA-seq)

Tumor and adjacent non-tumorous tissues were snap-frozen in liquid nitrogen and stored at −80 °C. Total RNA was extracted using TRIzol reagent, followed by DNase I treatment to remove genomic DNA contamination. RNA integrity was assessed using the Agilent 2100 Bioanalyzer. Samples meeting quality criteria underwent mRNA purification (for eukaryotic RNA) or rRNA depletion, followed by library preparation. Paired-end sequencing (PE150) was performed on the Illumina NovaSeq 6000 platform, with a minimum of 40 million reads per sample. Raw sequencing data underwent quality control with FastQC, and low-quality reads and adapter sequences were removed using Trimmomatic. Gene expression levels were quantified using featureCounts. Differentially expressed genes (DEGs) were identified using DESeq2 with thresholds of |log2FC| ≥ 1 and FDR < 0.05.

### Statistical analysis

Survival curves were generated using the Kaplan-Meier method, and differences between groups were compared using the log-rank test. Independent prognostic factors were identified using Cox proportional hazards regression models (reporting HR and 95% CI). Differences in WB and IHC results were analyzed using paired t-tests. Associations between CK19, Ki67, β-catenin expression, and clinicopathological features were evaluated using χ² tests or Fisher’s exact tests. A p-value < 0.05 was considered statistically significant. All statistical analyses were conducted using SPSS version 19.0.

## Results

### CK19, Ki67, and β-catenin are highly expressed in HCC tissues

**Fig. 1:**
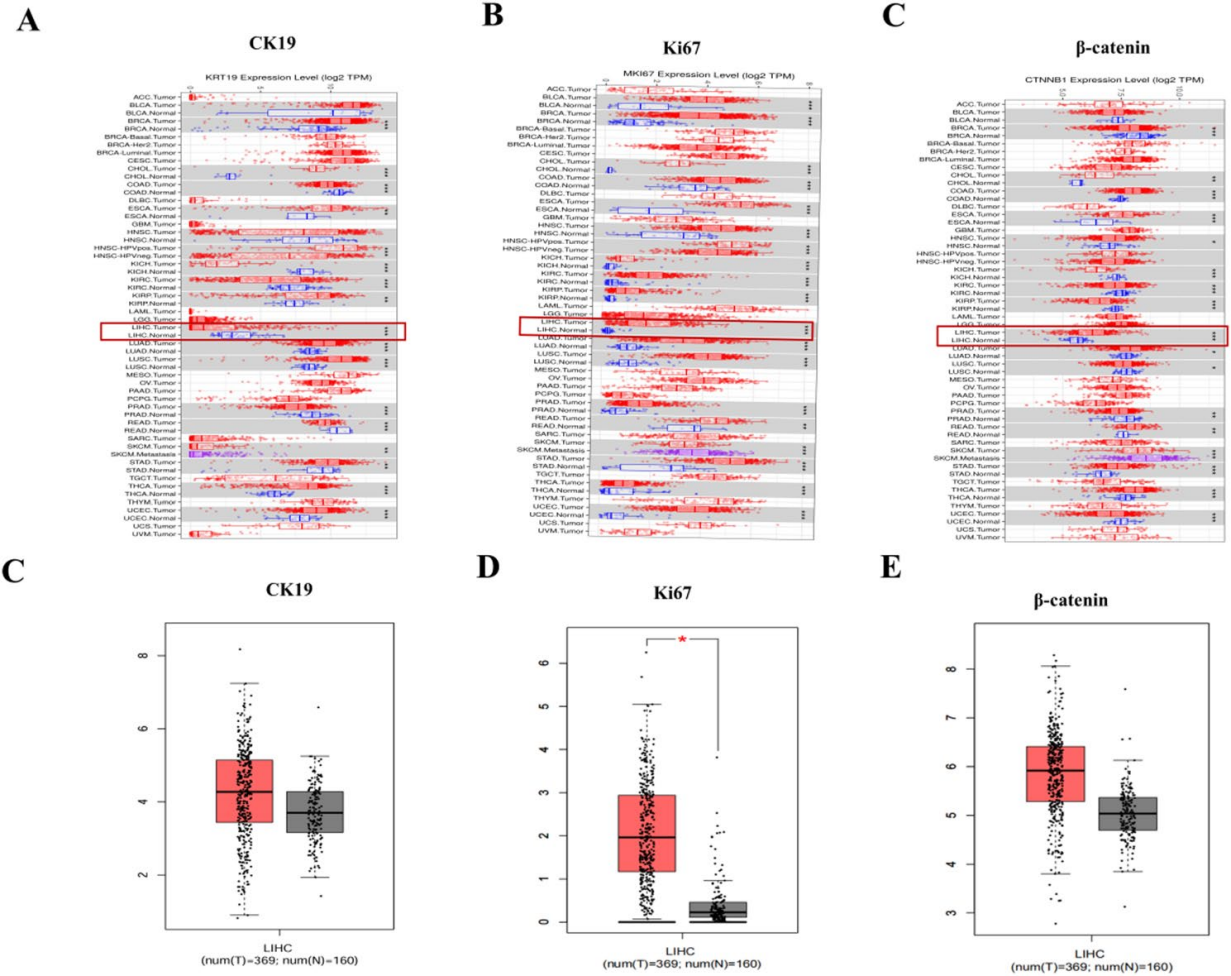
Expression levels of CK19, Ki67, and β-catenin in HCC tissues. **A**-**C** TIMER 2.0 database showing the expression of CK19 (KRT19), Ki67 (MKI67), and β-catenin (CTNNB1) across various tumor types. **D**-**E** GEPIA database showing expression levels of CK19 (KRT19), Ki67 (MKI67), and β-catenin (CTNNB1) in HCC and adjacent non-tumorous tissues

As mentioned before, CK19, Ki67, and β-catenin play critical roles in the development and progression of HCC. To further clarify their expression patterns in HCC tissues, we examined data from the TIMER 2.0 database. The results showed that CK19, Ki67, and β-catenin exhibited elevated expression trends across multiple tumor types. Specifically, in HCC tissues, both Ki67 and β-catenin were upregulated compared to adjacent non-tumorous tissues, with Ki67 showing statistically significant differences (Figs. 1A–C). Furthermore, we validated these findings using the GEPIA database, which integrates TCGA cancer datasets. The analysis revealed that CK19, Ki67, and β-catenin were highly expressed in HCC tissues compared to adjacent non-tumorous tissues (Figs. 1D–E). These results indicate a strong association between CK19, Ki67, β-catenin, and HCC.

### High expression levels of CK19, Ki67, and β-catenin are negatively correlated with patient survival

**Fig. 2:**
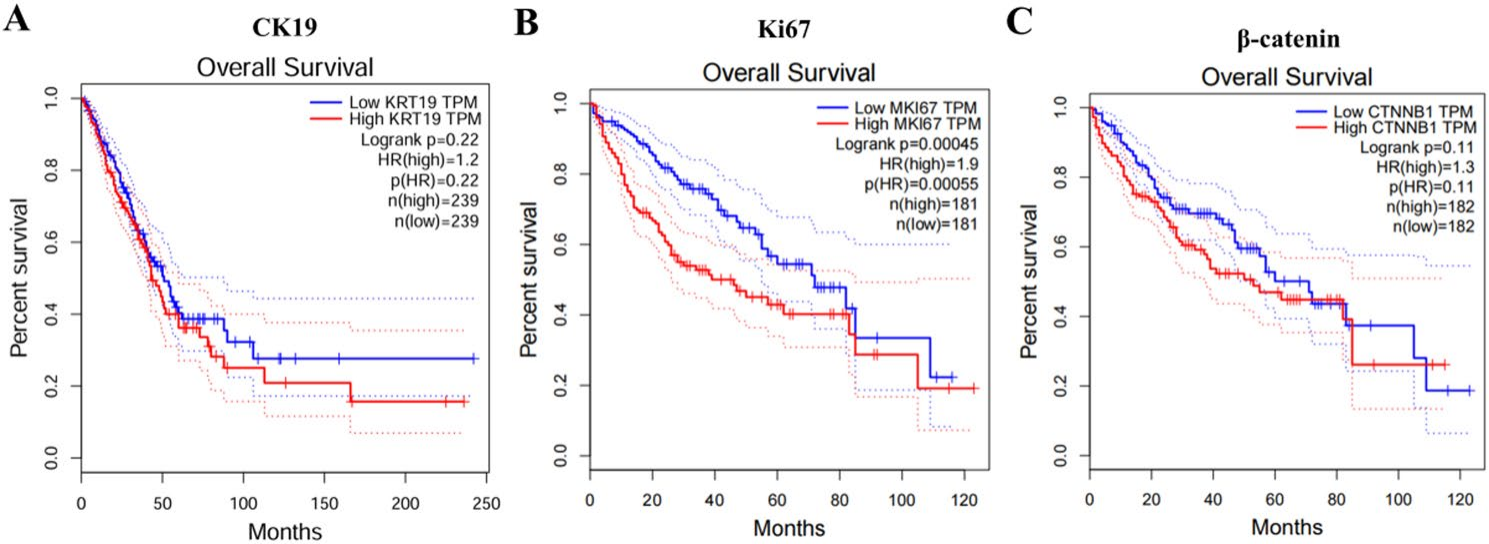
Prediction of patient survival based on CK19, Ki67, and β-catenin expression levels survival analysis from the GEPIA database

To investigate whether CK19, Ki67, and β-catenin are associated with poor prognosis in HCC patients’ post-surgery, we analyzed survival data from the GEPIA database. The results demonstrated that high expression of CK19 (KRT19), Ki67 (MKI67), and β-catenin (CTNNB1) was negatively correlated with overall survival (OS) in HCC patients, with higher expression levels corresponding to lower survival rates (Fig. 2).

**Fig. 3:**
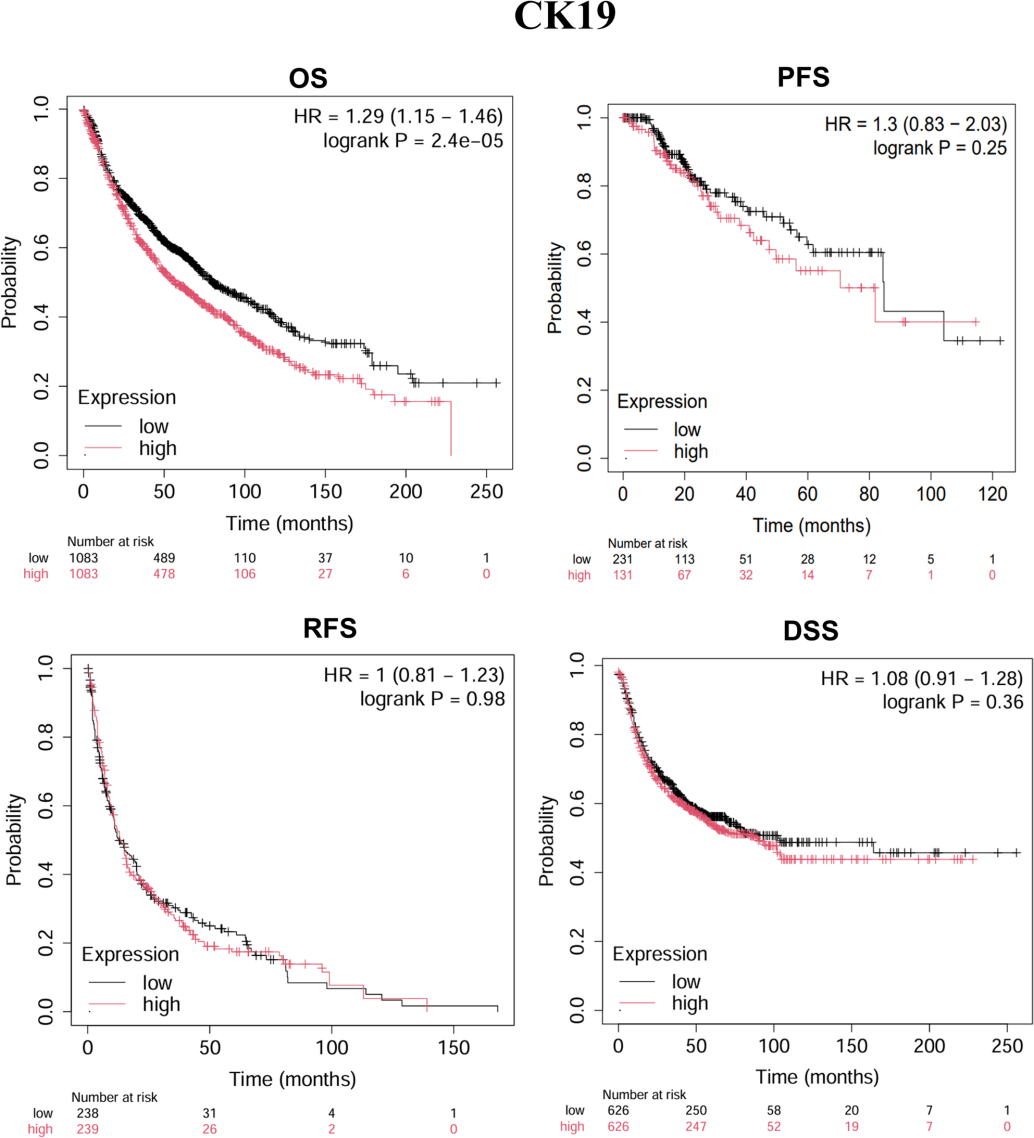
Kaplan-Meier analysis of CK19 expression in HCC patients post-surgery of OS, DSS, PFS, and RFS

**Fig. 4:**
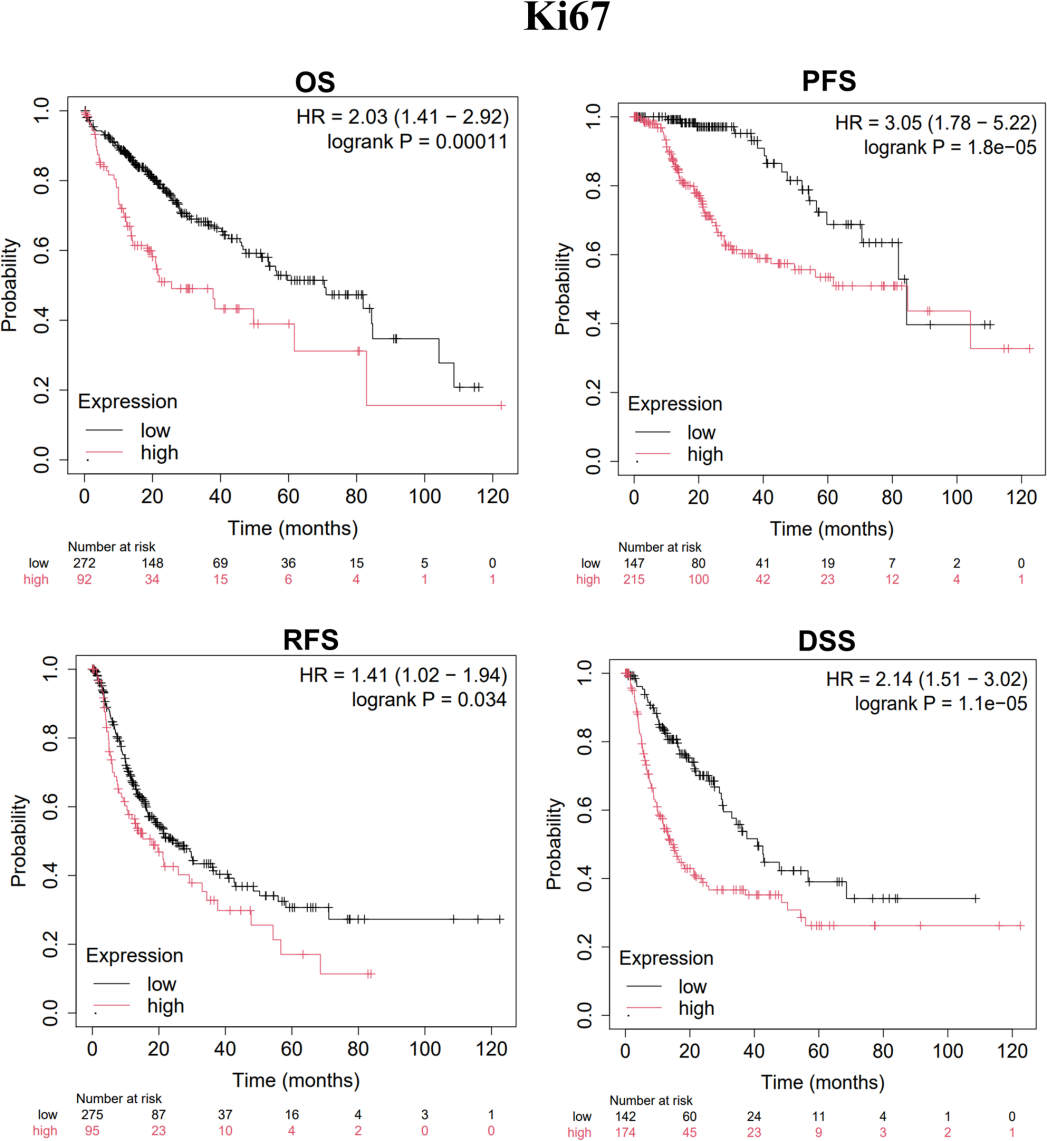
Kaplan-Meier analysis of Ki67 expression in HCC patients

**Fig. 5:**
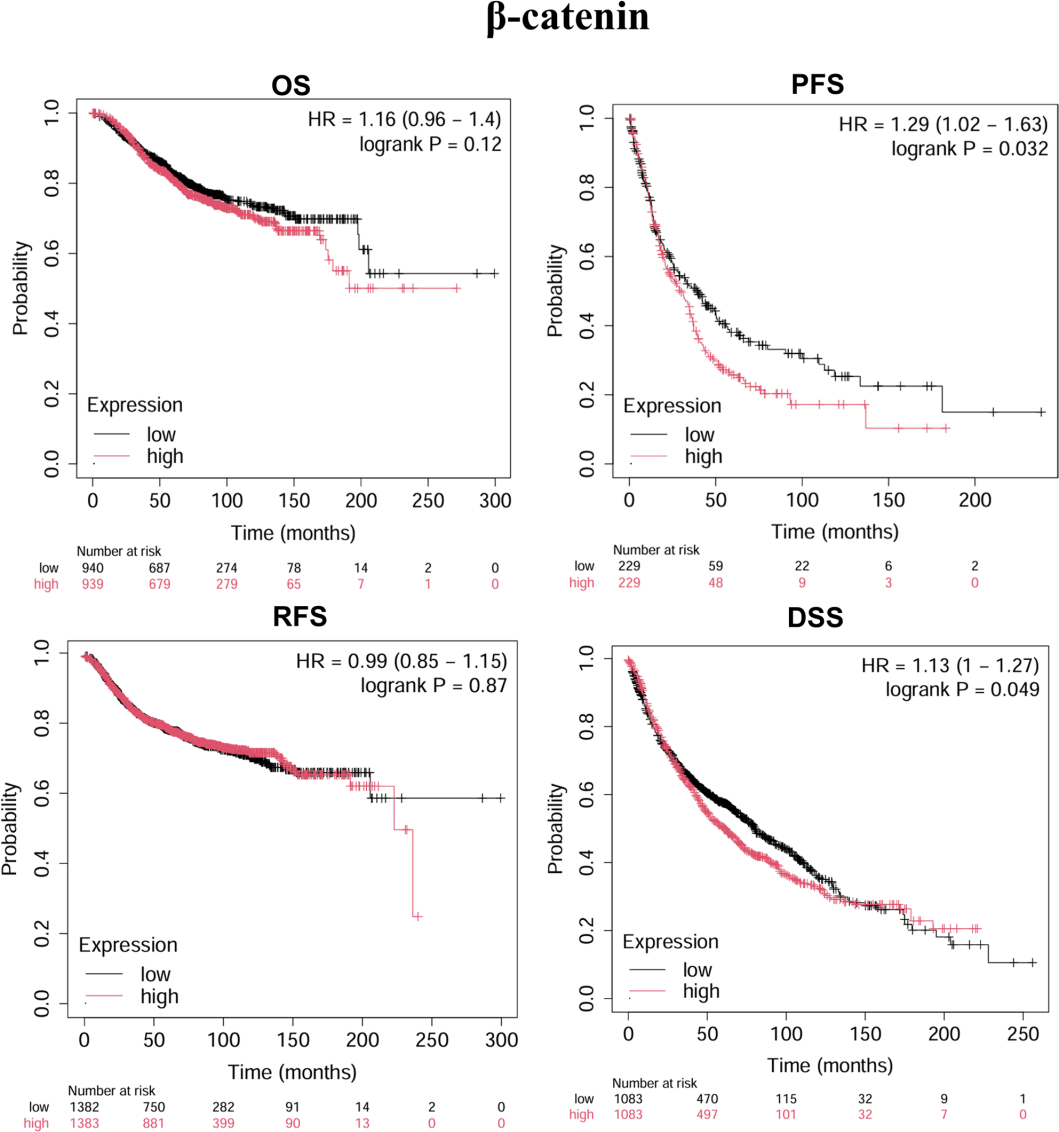
Kaplan-Meier analysis of β-catenin expression in HCC patients

Further analysis using the Kaplan-Meier Plotter database revealed that high CK19 expression was significantly associated with poor OS (*P* = 2.4e-05) (Fig. 3); high Ki67 expression was negatively correlated with OS (*P* = 0.00011), progression-free survival (PFS, *P* = 1.8e-05), recurrence-free survival (RFS, *P* = 0.034), and disease-specific survival (DSS, *P* = 1.1e-05) (Fig. 4); and high β-catenin expression was negatively associated with PFS (*P* = 0.032) and DSS (*P* = 0.049) (Fig. 5). These results suggest that CK19, Ki67, and β-catenin are independent adverse prognostic factors in HCC and may serve as independent biomarkers for prognosis.

### CK19, Ki67, and β-catenin are important prognostic factors in HCC

To determine the risk factors associated with DFS and OS, we comprehensively evaluated prognostic factors in HCC. Using univariate analysis and Cox regression models, we assessed 11 clinicopathological variables. For DFS, univariate analysis indicated that age, TNM stage, vascular invasion, liver cirrhosis, tumor capsule, lymphatic metastasis, tumor differentiation, and the expression of CK19, Ki67, and β-catenin were significant prognostic factors (all *P* < 0.05) (Table 1). Multivariate Cox regression analysis further confirmed that age, AFP, TNM stage, liver cirrhosis, tumor capsule, and the expression levels of CK19, Ki67, and β-catenin were independent prognostic factors (Table 2). Similarly, for OS, univariate analysis identified age, AFP serum levels, TNM stage, vascular invasion, tumor capsule, lymphatic metastasis, tumor differentiation, and the expression of CK19, Ki67, and β-catenin as important factors (all *P* < 0.05) (Table 2). Multivariate Cox analysis indicated that age, AFP, liver cirrhosis, tumor capsule, lymphatic metastasis, tumor differentiation, and the expression of CK19, Ki67, and β-catenin were independent prognostic factors (Table 3). Together, these results suggest that CK19, Ki67, and β-catenin are crucial prognostic markers closely related to both DFS and OS in HCC patients.

**Table 1:** Prognostic factors for DFS and OS by univariate analysis

	DFS				OS		
Variables	*n*	1-yr	3-yrs	*P*	1-yr	3-yrs	*P*
Gender							
Male	75	40%	30%	0.419	77%	49%	0.625
Female	35	49%	30%		76%	44%	
Age (yrs)							
≤ 60	48	42%	27%	0.053	74%	44%	0.035
> 60	62	65%	45%		95%	65%	
Tumor size							
≦ 5.0	66	46%	23%	0.609	92%	46%	0.909
≦ 5.0	44	45%	30%		75%	47%	
Tumor number							
1	89	42%	29%	0.476	76%	47%	0.476
≧ 2	21	61%	30%		78%	48%	
AFP							
> 400	37	47%	30%	0.229	78%	50%	0.067
≦ 400	83	33%	24%		62%	29%	
TNM staging							
I	46	46%	27%	0.359	74%	44%	0.022
II	21	33%	37%		86%	57%	
III	15	62%	42%		88%	60%	
I	28	37%	24%		71%	41%	
Vascular invasion							
Yes	42	53%	33%	0.001	80%	55%	<.001
No	68	27%	14%		67%	29%	
Cirrhosis							
Yes	85	10%	3%	< 0.001	40%	13%	<.001
No	25	53%	35%		85%	55%	
Tumor encapsulation							
Yes	73	57%	35%	< 0.001	50%	33%	0.001
No	39	24%	20%		59%	34%	
Lymphatic metastasis							
Yes	25	50%	33%	0.016	50%	33%	0.016
No	85	29%	16%		30%	16%	
Differentiation							
Low	25	32%	30%	0.0244	35%	32%	0.021
Medium	62	24%	21%		22%	12%	
High	23	13%	5%		4%	6%	
CK19 expression							
Low	48	10%	3%	< 0.001	40%	13%	<.001
High	62	53%	35%		85%	55%	
Ki67 expression							
Low	27	50	33	0.002	81%	52%	<.001
High	73	23	13		57%	23%	
β-catenin expression							
Low	21	31%	13%	0.002	65%	29%	<.001
High	89	51%	37%		81%	55%	

*DFS* Disease-free survival, *HCC* hepatocellular carcinoma, *OS* overall survival, resection margin, the nearest distance between tumor and the resection plan

**Table 2:** Prognostic factors for disease-free and overall survival by the multivariate Cox proportional hazards regression model

	DFS			OS		
Variables	HR	95% CI	*P*	HR	95% CI	*P*
Gender				1.007	0.990–1.025	0.428
Age	2.051	0.920–4.572	0.079	2.068	1.002–4.863	0.065
Tumor size	1.184	0.759–1.849	0.457	1.063	0.624–1.810	0.823
Tumor number	1.395	0.744–2.615	0.299	1.385	0.711–2.695	0.338
AFP	2.695	1.610–4.511	< 0.001	2.645	1.570–4.457	< 0.001
TNM staging	0.691	0.446–1.071	0.098	0.689	0.418–1.134	0.143
CK19 expression	0.469	0.303–0.727	0.001	0.435	0.257–0.736	0.002
Ki67 expression	0.369	0.287–0.678	0.001	0.321	0.213–0.638	0.001
β-catenin expression	0.543	0.454–0.823	0.001	0.532	0.324–0.826	0.001
Vascular invasion	1.049	0.658–1.673	0.840	0.999	0.614–1.623	0.995
Cirrhosis	1.573	1.009–2.451	0.046	2.079	1.274–3.392	0.003
Tumor encapsulation	0.469	0.303–0.727	0.001	0.435	0.257–0.736	0.002
Lymphatic metastasis	0.781	0.403–1.514	0.464	0.733	0.351–1.532	0.409
Differentiation	1.410	0.839–2.372	0.195	1.697	0.933–3.088	0.083

**Table 3:** Correlation between the expression of CK19, Ki67, and β-catenin and the clinicopathological characteristics of HCC patients

Clinicopathological characteristics		CK19 expression			Ki67 expression			β-catenin expression		
		-	+	*P*	-	+	*P*	-	+	*P*
Gender				1.000 ∗			1.000 ∗			0.238 ∗
	Male	35	40		50	37		49	40	
	Female	25	10		12	11		3	18	
Age				0.388 ∗			0.088 ∗			1.000 ∗
	≦ 60	27	21		46	33		41	43	
	> 60	33	29		16	15		13	13	
Tumor size				0.203 ∗			0.037 ∗			0.044 ∗
	≦ 5.0	36	30		36	20		33	22	
	> 5.0	24	20		26	28		20	35	
Tumor number				0.005 ∗			0.005 ∗			0.617 ∗
	1	49	40		21	35		26	25	
	≧ 2	11	10		36	18		28	31	
AFP				0.174 ∗			1.000 ∗			0.419 ∗
	> 400	16	21		23	18		18	22	
	≦ 400	44	29		40	29		36	34	
TNM staging				0.122 ∗			0.659 ∗			0.857 ∗
	I	34	12		10	13		5	6	
	II	11	10		22	14		9	21	
	III	10	5		6	10		14	10	
	IV	5	23		23	22		23	22	
Vascular invasion				0.398 ∗			0.783 ∗			0.544 ∗
	Yes	24	18		16	22		7	11	
	No	36	32		41	31		22	20	
Cirrhosis				0.072 ∗			0.379 ∗			0.082 ∗
	Yes	53	32		38	38		25	21	
	No	8	17		19	15		4	10	
Tumor encapsulation				0.416 ∗			0.578 ∗			0.022 ∗
	Yes	45	26		39	34		40	33	
	No	23	16		18	19		19	18	
Lymphatic metastasis				1.000 ∗			0.025 ∗			0.750 ∗
	Yes	20	5		10	12		10	12	
	No	49	36		47	41		44	44	
Differentiation				0.038 ∗			0.035 ∗			0.081 ∗
	Low	15	10		7	15		5	10	
	Medium	42	20		37	28		31	47	
	High	14	9		14	8		5	12	

### CK19, Ki67, and β-catenin protein levels are significantly elevated in HCC tissue samples

**Fig. 6:**
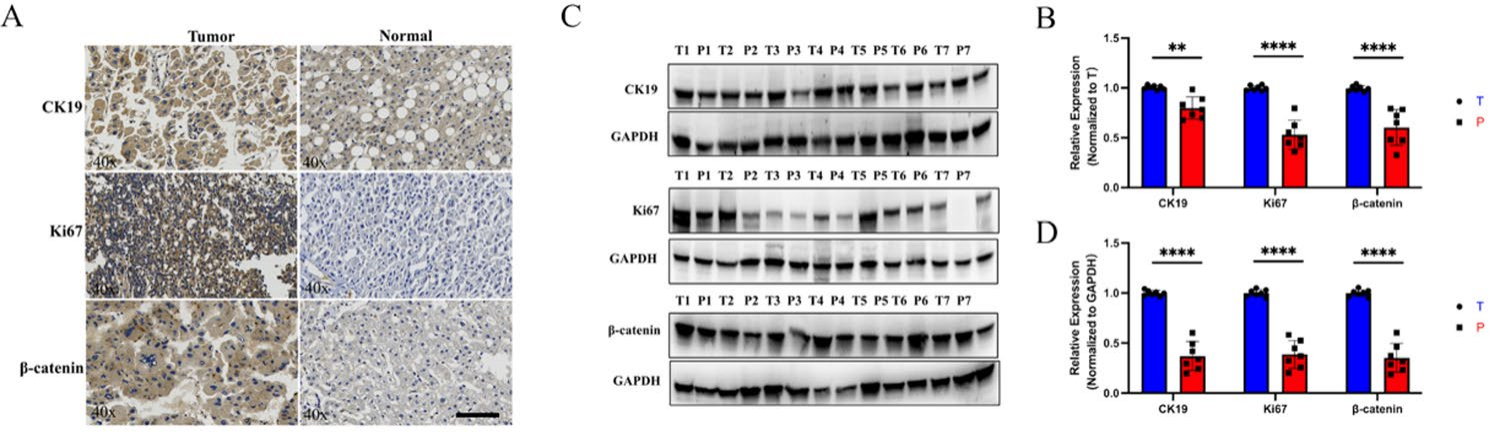
Expression levels of CK19, Ki67, and β-catenin in HCC and adjacent tissues. **A**-**B** IHC staining. Scale bar: 100 μm. **C**-**D** Western blot analysis of seven paired HCC and adjacent tissues (T: tumor tissue; P: paired non-tumorous tissue)

To further validate changes in CK19, Ki67, and β-catenin in HCC, we examined pathological samples from HCC patients. IHC staining revealed that CK19, Ki67, and β-catenin were highly expressed in tumor tissues compared to adjacent non-tumorous tissues. Additionally, WB analyses showed significantly increased protein levels of CK19, Ki67, and β-catenin in tumor tissues (Fig. 6). These findings support the conclusion that CK19, Ki67, and β-catenin are upregulated in HCC and are linked to disease progression.

### Transcriptomic sequencing suggests upregulation of metabolic reprogramming, immune evasion, and invasive phenotypes in HCC

**Fig. 7:**
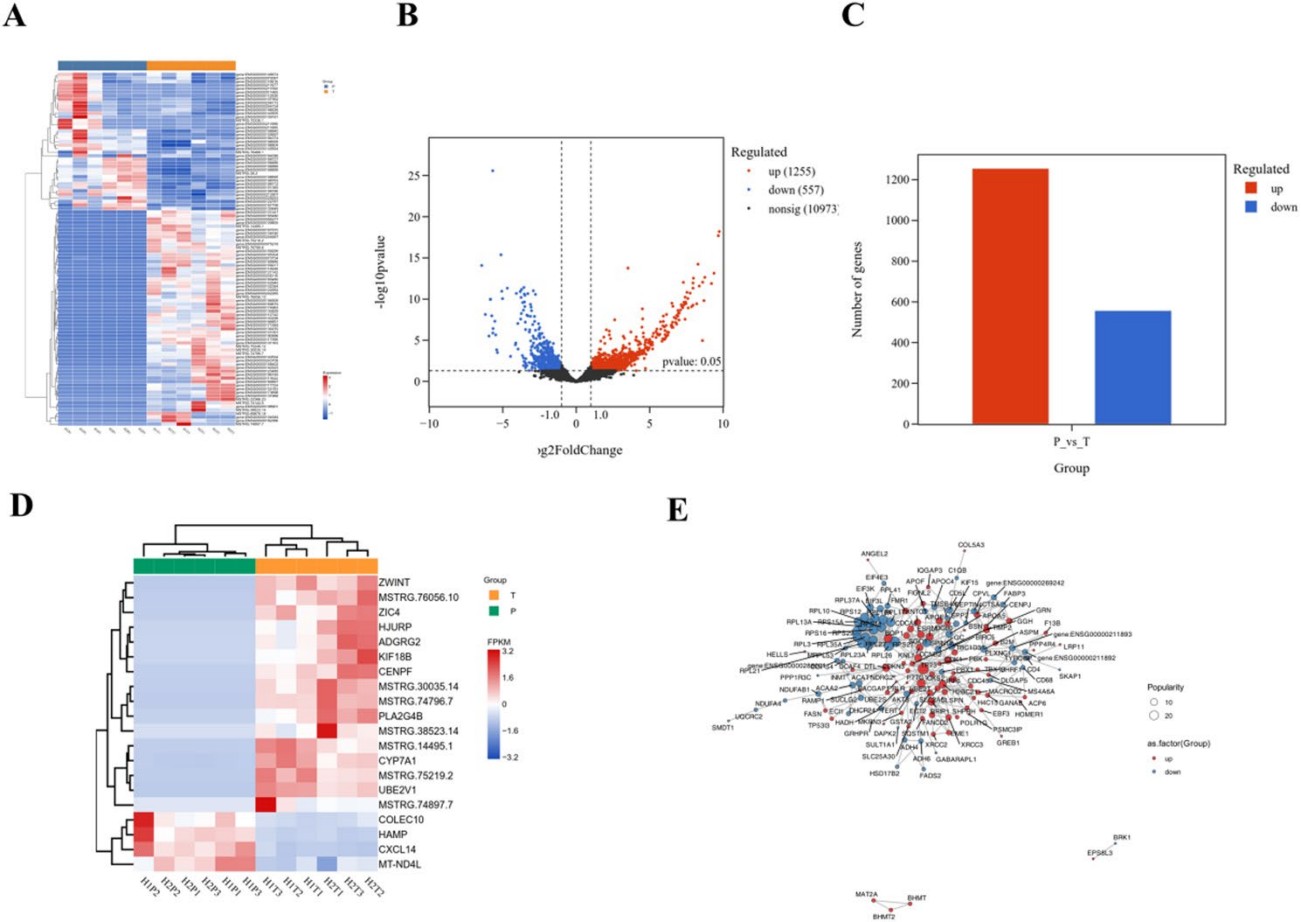
Transcriptomic analysis of tumor and adjacent tissues. **A** Heatmap of differential gene expression. **B** Volcano plot of differentially expressed genes. **C **Gene count statistics. **D** Heatmap of oncogenes and tumor suppressor genes. **E** Protein-protein interaction network analysis

To elucidate changes during HCC progression, we performed RNA-seq on tumor and adjacent non-tumorous liver tissues. Heatmap analysis showed significant transcriptional differences between HCC and adjacent tissues (Fig. 7A). Differential gene analysis (|log2FC| ≥ 1 and FDR < 0.05) identified 1,255 upregulated and 557 downregulated genes (Figs. 7B–C). Further analysis of oncogenes and tumor suppressor genes revealed that tumors exhibited elevated expression of oncogenes such as ZWINT and ZIC4, and decreased expression of tumor suppressors like HAMP and CXCL14 (Fig. 7D). Protein-protein interaction (PPI) network analysis highlighted key drivers including ZWINT (chromosome segregation regulation), IQGAP3 (invasion and migration promotion), MAT2A (SAMe synthesis), C1QB (complement pathway), and ANGEL2 (RNA metabolism regulation) (Fig. 7E). Overall, the upregulation of oncogenes and downregulation of tumor suppressors contribute to tumor proliferation, metabolic reprogramming, immune escape, and metastatic phenotypes in HCC.

### Expression of CK19, Ki67, and β-catenin correlates with pathological features associated with disease progression

To better understand the clinical significance of CK19, Ki67, and β-catenin in HCC progression, we analyzed their association with 11 clinicopathological features (Table III). Results indicated that CK19 expression was significantly associated with tumor number (*P* = 0.005) and tumor differentiation grade (*P* = 0.038), but not with other clinical features. Ki67 expression was closely related to patient age (*P* = 0.088), tumor size (*P* = 0.037), tumor number (*P* = 0.005), lymph node metastasis (*P* = 0.025), and tumor differentiation (*P* = 0.035). β-catenin expression correlated with tumor diameter (*P* = 0.044), tumor number, and capsule integrity (*P* = 0.022). Specifically, CK19 and Ki67 were significantly associated with tumor multifocality, suggesting a link between stem-like features, high proliferation, and aggressive behavior. Ki67 and β-catenin expression levels were related to tumor size, indicating stronger proliferative capacity in larger tumors and possibly higher malignancy. Moreover, CK19 and Ki67 were associated with poorer differentiation, while Ki67 was linked to lymph node metastasis, reflecting enhanced proliferative and metastatic abilities. β-catenin expression was higher in tumors lacking capsule formation, suggesting a role in promoting invasiveness. In conclusion, CK19 correlates with stemness/multifocality, Ki67 reflects proliferative activity, and β-catenin is associated with aggressive progression in HCC.

## Discussion

In this study, by integrating bioinformatics analysis, immunohistochemical detection, transcriptomic sequencing analysis, and clinicopathological correlation studies, we systematically revealed the expression characteristics and prognostic value of CK19, Ki67, and β-catenin in HCC. Our results showed that all three markers were significantly upregulated in HCC tissues and closely associated with poor prognosis and higher mortality in HCC patients. Furthermore, analysis of the relationship between these markers and clinicopathological features demonstrated that CK19, Ki67, and β-catenin expression levels were significantly correlated with key malignant features of HCC, including differentiation degree, multifocality, lymphatic metastasis, and liver cirrhosis, which were further validated by transcriptomic sequencing results. In contrast to previous studies focusing on individual markers, this study integrates multi-dimensional analyses, including bioinformatics, immunohistochemistry, transcriptomic profiling, and clinicopathological correlation, to comprehensively elucidate the expression patterns and prognostic implications of CK19, Ki67, and β-catenin in HCC. This integrative approach highlights the novel insight that the combined expression of these markers contributes to HCC heterogeneity and aggressiveness, offering a valuable foundation for biomarker discovery and therapeutic target development.

Previous studies have confirmed that CK19 expression is elevated in liver injury and [22] HCC tissues [23]. Consistently, our database analysis, immunohistochemical staining, and WB experiments verified that CK19 levels were significantly higher in HCC tissues compared with adjacent non-tumorous tissues. High CK19 expression has been associated with poor prognosis, particularly with shortened recurrence-free survival (RFS) [24]. In our study, high CK19 expression was also significantly negatively correlated with overall survival (OS). Moreover, CK19 has been implicated in regulating cancer stem cell properties and remodeling the tumor microenvironment [25]. The development of efficient fluorescent probes for detection and imaging is of particular importance [26]. Our analysis further demonstrated that CK19 expression was significantly associated with tumor number and differentiation degree, suggesting a strong link between CK19 expression and tumor multifocality, stem-like properties, and proliferative potential. Ki67 is a well-established marker of cell proliferation and has been shown to be highly expressed in HCC tissues [27–29], with high expression significantly worsening overall survival rates [30]. In our analysis, Ki67 overexpression was correlated with OS, PFS, RFS, and DSS, underscoring its critical role as a core indicator of proliferative activity and tumor aggressiveness. Notably, elevated Ki67 proliferation indices in peritumoral hepatocytes may also provide molecular evidence for surgical margin evaluation during liver resection [31]. β-catenin, a key component of the Wnt/β-catenin signaling pathway, has been closely associated with disease progression and increased risk of cancer-specific mortality in HCC tissue and in vitro model [32]. Through the activation of epithelial-mesenchymal transition and vascular invasion, β-catenin promotes HCC invasiveness and metastasis [33]. Abnormal β-catenin expression is associated with elevated serum alpha-fetoprotein (AFP) levels, poor tumor differentiation, and vascular invasion. Furthermore, increased AFP levels have been shown to upregulate β-catenin, suggesting a strong interplay between β-catenin dysregulation and HCC pathogenesis [34]. In our study, we similarly observed concurrent upregulation of AFP and β-catenin, both significantly associated with patient survival. Establishment of diagnostic and predictive models can to some extent guide clinical treatment and improve patient prognosis [27, 35].

The novelty of this study lies in the first systematic investigation of the alterations of CK19, Ki67, and β-catenin in HCC. By combining public database analyses and validation with clinical patient samples, we confirmed the upregulation of these three molecules in HCC and their close association with survival outcomes. Univariate and Cox regression analyses further confirmed that all three markers are independent prognostic factors for HCC. In addition, the expression levels of CK19, Ki67, and β-catenin were significantly associated with multiple clinicopathological features of HCC progression, offering new perspectives for multi-gene prognostic modeling and multi-pathway targeted therapy development.

However, this study has some limitations. Firstly, the retrospective design may introduce selection bias. And other liver-related systemic diseases may have influenced the interpretation of our results [36, 37]. Secondly, the relatively small sample size may limit the generalizability of the findings. Future studies with larger cohorts, prospective validation, and molecular mechanism investigations are needed to construct a comprehensive prognostic model integrating clinicopathological features with multi-omics biomarkers, providing more precise strategies for individualized HCC treatment and recurrence monitoring. Moreover, this study focused primarily on the individual relationship between each molecule and patient mortality without exploring the combined diagnostic and prognostic efficacy of the three markers. Future research will aim to build integrated models assessing the combined predictive value of CK19, Ki67, and β-catenin. Additionally, the study did not delve into the interaction networks among the three molecules or their dynamic association with the immune microenvironment, which will be important directions for subsequent research focusing on their roles in tumor immunity and immunomodulation. And multiple molecular factors contributed to HCC progression and influence clinical outcomes should be concluded [38, 39].

## Conclusion

This study confirmed that CK19, Ki67, and β-catenin are significantly overexpressed in HCC tissues, and collectively drive postoperative recurrence of HCC. The combined detection of these three molecular markers can overcome the limitations of traditional AFP-based monitoring, providing a high-sensitivity and high-specificity recurrence warning model for AFP-negative HCC patients. Furthermore, these markers suggest potential therapeutic targets, ultimately contributing to improved survival outcomes for HCC patients.

## Supplementary Information

Supplementary Material 1.

## Data Availability

The datasets generated and/or analyzed during the current study are available from the corresponding author on reasonable request.The datasets generated and analysed during the current study are available in the NCBI BioProject repository, under accession number PRJNA1369736.

## Abbreviations

AFP: Alpha-fetoprotein
CK19: Cytokeratin 19
DFS: Disease-free survival
DSS: Disease-specific survival
HCC: Hepatocellular carcinoma
HR: Hazard ratio
IHC: Immunohistochemistry
OS: Overall survival
PFS: Progression-free survival
RFS: Recurrence-free survival
WB: Western blotting

## References

[1] Hepatocellular carcinoma. Nat Rev Dis Primers. 2021;7(1):7. 33479233 10.1038/s41572-021-00245-6

[2] Singal AG, Kanwal F, Llovet JM. Global trends in hepatocellular carcinoma epidemiology: implications for screening, prevention and therapy. Nat Rev Clin Oncol. 2023;20(12):864–84. 37884736 10.1038/s41571-023-00825-3

[3] Chakraborty E, Sarkar D. Emerging therapies for hepatocellular carcinoma (HCC). Cancers. 2022;14(11):2798. 35681776 10.3390/cancers14112798 PMC9179883

[4] Clark T, Maximin S, Meier J, et al. Hepatocellular carcinoma: review of epidemiology, screening, imaging diagnosis, response assessment, and treatment. Curr Probl Diagn Radiol. 2015;44(6):479–86. 25979220 10.1067/j.cpradiol.2015.04.004

[5] Chen LT, Martinelli E, Cheng AL, et al. Pan-Asian adapted ESMO clinical practice guidelines for the management of patients with intermediate and advanced/relapsed hepatocellular carcinoma: a TOS-ESMO initiative endorsed by CSCO, ISMPO, JSMO, KSMO, MOS and SSO. Ann Oncol. 2020;31(3):334–51. 32067677 10.1016/j.annonc.2019.12.001

[6] Johnson P, Zhou Q, Dao DY, et al. Circulating biomarkers in the diagnosis and management of hepatocellular carcinoma. Nat Rev Gastroenterol Hepatol. 2022;19(10):670–81. 35676420 10.1038/s41575-022-00620-y

[7] Koch C, Bette T, Waidmann O, et al. AFP ratio predicts HCC recurrence after liver transplantation. PLoS One. 2020;15(7):e0235576. 32614912 10.1371/journal.pone.0235576 PMC7332004

[8] Shao M, Tao Q, Xu Y, et al. Glutamine synthetase-negative hepatocellular carcinoma has better prognosis and response to sorafenib treatment after hepatectomy. Chin Med J (Engl). 2023;136(17):2066–76. 37249521 10.1097/CM9.0000000000002380 PMC10476731

[9] Yang X, Ni H, Lu Z, et al. Mesenchymal Circulating tumor cells and Ki67: their mutual correlation and prognostic implications in hepatocellular carcinoma. BMC Cancer. 2023;23(1):10. 36600214 10.1186/s12885-023-10503-3 PMC9814317

[10] Ruiz de Galarreta M, Bresnahan E, Molina-Sánchez P, et al. β-Catenin activation promotes immune escape and resistance to Anti-PD-1 therapy in hepatocellular carcinoma. Cancer Discov. 2019;9(8):1124–41. 31186238 10.1158/2159-8290.CD-19-0074 PMC6677618

[11] Shuyao W, Mingyang B, Feifei M, et al. CK19 predicts recurrence and prognosis of HBV positive HCC. J Gastrointest Surgery. 2022;26(2):341–51. 10.1007/s11605-021-05107-w 34506016

[12] Feng J, Zhu R, Chang C, et al. CK19 and glypican 3 expression profiling in the prognostic indication for patients with HCC after surgical resection. PLoS One. 2016;11(3):e0151501. 26977595 10.1371/journal.pone.0151501 PMC4792431

[13] Yu J-P, Xu X-G, Ma R-J, et al. Development of a clinical chemiluminescent immunoassay for serum GPC3 and simultaneous measurements alone with AFP and CK19 in diagnosis of hepatocellular carcinoma. J Clin Lab Analysis. 2015;29(2):85–93. 10.1002/jcla.21733 PMC6806998 24687454

[14] Lee C-W, Lin S-E, Yu M-C, et al. Does neutrophil to lymphocyte ratio have a role in identifying cytokeratin 19-Expressing hepatocellular carcinoma? J Personalized Medicine. 2021;11(11):1078. 10.3390/jpm11111078 PMC8621990 34834430

[15] Zhang X, Wu Z, Peng Y, et al. Correlationship between Ki67, VEGF, and p53 and hepatocellular carcinoma recurrence in liver transplant patients. BioMed Res International. 2021;2021(1):6651397. 10.1155/2021/6651397 PMC8064788 33954191

[16] Schmitt-Graeff A, Ertelt-Heitzmann V, Allgaier H-P, et al. Coordinated expression of Cyclin D1 and LEF-1/TCF transcription factor is restricted to a subset of hepatocellular carcinoma. Liver International. 2005;25(4):839–47. 15998435 10.1111/j.1478-3231.2005.01069.x

[17] Espírito Santo J, Ladeirinha A, Alarcão A, et al. Hepatocellular carcinoma: tumor heterogeneity and recurrence after preoperative locoregional therapy. Med Oncol. 2023;40(12):340. 37882867 10.1007/s12032-023-02208-1

[18] Deldar Abad Paskeh M, Mirzaei S, Ashrafizadeh M, et al. Wnt/β-Catenin signaling as a driver of hepatocellular carcinoma progression: an emphasis on molecular pathways. J Hepatocell Carcinoma. 2021;8:1415–44. 34858888 10.2147/JHC.S336858 PMC8630469

[19] Xu A, Wang X, Luo J, et al. Overexpressed P75CUX1 promotes EMT in glioma infiltration by activating β-catenin. Cell Death Dis. 2021;12(2):157. 33542188 10.1038/s41419-021-03424-1 PMC7862635

[20] Pang Q, Hu W, Zhang X, et al. Wnt/β-catenin signaling pathway-related proteins (DKK-3, β-catenin, and c-MYC) are involved in prognosis of nasopharyngeal carcinoma. Cancer Biother Radiopharm. 2019;34(7):436–43. 31025872 10.1089/cbr.2019.2771

[21] Xu Y, Yu X, Sun Z, et al. Roles of lncRNAs mediating Wnt/β-catenin signaling in HCC. Front Oncol. 2022;12:831366. 35356220 10.3389/fonc.2022.831366 PMC8959654

[22] Chen F, Zhang K, Wang M, et al. VEGF-FGF signaling activates quiescent CD63(+) liver stem cells to proliferate and differentiate. Adv Sci (Weinh). 2024;11(33):e2308711. 38881531 10.1002/advs.202308711 PMC11434209

[23] Govaere O, Komuta M, Berkers J, et al. Keratin 19: a key role player in the invasion of human hepatocellular carcinomas. Gut. 2014;63(4):674–85. 23958557 10.1136/gutjnl-2012-304351 PMC3963546

[24] Rhee H, Kim H, Park YN. Clinico-radio-pathological and molecular features of hepatocellular carcinomas with keratin 19 expression. Liver Cancer. 2020;9(6):663–81. 33442539 10.1159/000510522 PMC7768132

[25] Yang C-L, Song R, Hu J-W, et al. Integrating single-cell and bulk RNA sequencing reveals CK19 + cancer stem cells and their specific SPP1 + tumor-associated macrophage niche in HBV-related hepatocellular carcinoma. Hepatol Int. 2024;18(1):73–90. 38159218 10.1007/s12072-023-10615-9

[26] Men X, Liu F, Gong M, et al. Activatable fluorescent probes for imaging and diagnosis of hepatocellular carcinoma. J Innovative Opt Health Sciences. 2025;18(03):2530004.

[27] Liu X, Jiang D, Liu Y, et al. Crispr-Cas9-based long non-coding RNA interference and activation identified that the aberrant expression of Myc-regulated ST8SIA6 antisense RNA 1 promotes tumorigenesis and metastasis in hepatocellular carcinoma. Cytojournal. 2024;21:53. 39737136 10.25259/Cytojournal_109_2024 PMC11683396

[28] Peng X, Liu Z, Luo C, et al. PSMD12 promotes hepatocellular carcinoma progression by stabilizing CDK1. Front Immunol. 2025;16:1581398. 40534847 10.3389/fimmu.2025.1581398 PMC12174133

[29] Bai K, Cao Y, Huang Q, et al. Prognostic value of Ki67 expression for patients with surgically resected hepatocellular carcinoma: perspectives from a high incidence area. Clin Lab. 2017;63(2):355–64. 28182353 10.7754/Clin.Lab.2016.160638

[30] Lei HJ, Wang SY, Chau IY, et al. Hepatoma upregulated protein and Ki-67 expression in resectable hepatocellular carcinoma. J Chin Med Assoc. 2021;84(6):623–32. 33883465 10.1097/JCMA.0000000000000540 PMC12966124

[31] Farris AB 3rd, Dursun N, Dhanasekaran R, et al. Tumoral and angiogenesis factors in hepatocellular carcinoma after locoregional therapy. Pathol Res Pract. 2012;208(1):15–21. 22088254 10.1016/j.prp.2011.10.005

[32] Chen F, Wang Z, Yao H, et al. Large-scale manufacturing of human gallbladder epithelial cell products and derived hepatocytes via a chemically defined approach. Trends Biotechnol. 2025;43(10):2646–64. 40399214 10.1016/j.tibtech.2025.04.009

[33] Tian Y, Xia J, Yang G, et al. A2aR inhibits fibrosis and the EMT process in silicosis by regulating Wnt/β-catenin pathway. Ecotoxicol Environ Saf. 2023;249:114410. 36516619 10.1016/j.ecoenv.2022.114410

[34] Ren YJ, Huang T, Yu HL, et al. Expression of β-catenin protein in hepatocellular carcinoma and its relationship with alpha-fetoprotein. J Huazhong Univ Sci Technolog Med Sci. 2016;36(6):846–51. 27924522 10.1007/s11596-016-1673-9

[35] Liu G, Long J, Liu C, et al. Development and verification of a nomogram for predicting portal vein tumor thrombosis in hepatocellular carcinoma. Am J Transl Res. 2024;16(12):7511–20. 39822560 10.62347/PLQF5135 PMC11733390

[36] Ning Q, Yang T, Guo X, et al. CHB patients with rtA181T-mutated HBV infection are associated with higher risk hepatocellular carcinoma due to increases in mutation rates of tumour suppressor genes. J Viral Hepatitis. 2023;30(12):951–8. 10.1111/jvh.13886 37735836

[37] Zarlashat Y, A T, Hussain S. Neutrophil-to-lymphocyte and platelet-to-lymphocyte ratios in hepatocellular carcinoma: from inflammation to clinical applications. CP. 2024. 10.36922/cp.5758.

[38] Zhidan L, Yongxia Z. GSK-126 enhances all-trans-retinoic acid (ATRA) response in hepatocellular carcinoma (HCC) by upregulating < i > RARG</i > expression. Discov Med. 2024;36(184):1041–53. 38798263 10.24976/Discov.Med.202436184.97

[39] Liu B, Li C, He S, et al. Ubiquitin-conjugating enzyme E2S (UBE2S) as a prognostic biomarker and regulator of tumorigenesis in osteosarcoma. Int Immunopharmacology. 2025;154:114545. 10.1016/j.intimp.2025.114545 40188527

