# Supplementary figures and images for: Elevated expression of CK19, Ki67, and β-Catenin as prognostic biomarkers in hepatocellular carcinoma
Source: BMC Cancer. 2025 Dec 12;26:96. doi: 10.1186/s12885-025-15429-6 (PMC12821990; doi:10.1186/s12885-025-15429-6)

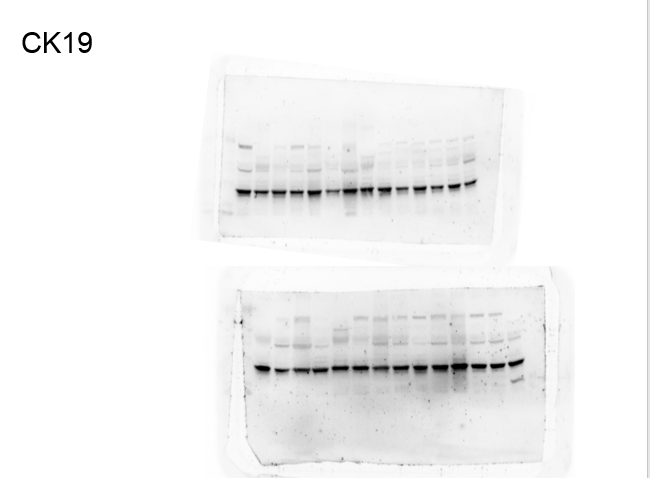


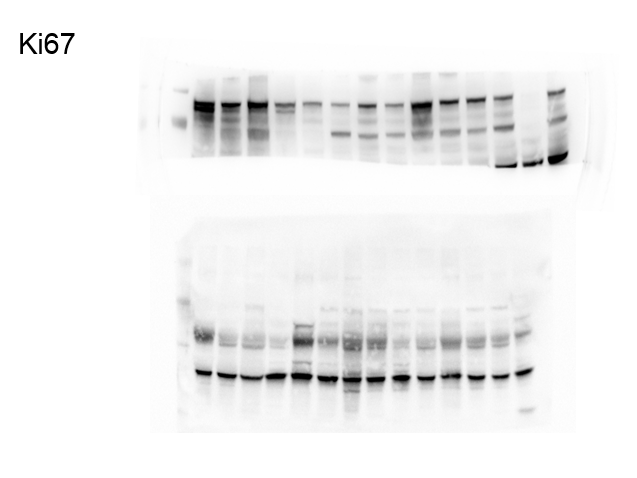


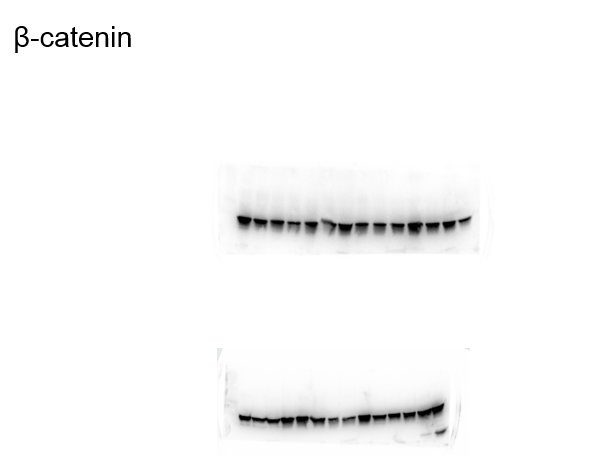

Supplement: Supplementary file 1 — Supplementary Material 1. [file 12885_2025_15429_MOESM1_ESM.docx]
